# The impact of dietary protein supplementation on recovery from resistance exercise-induced muscle damage: A systematic review with meta-analysis

**DOI:** 10.1038/s41430-022-01250-y

**Published:** 2022-12-13

**Authors:** Alice G. Pearson, Karen Hind, Lindsay S. Macnaughton

**Affiliations:** 1 Department of Sport and Exercise Sciences, Durham University, Durham, UK; 2 Wolfson Research Institute for Health and Wellbeing, Durham University, Durham, UK

**Keywords:** Physiology, Systems biology

## Abstract

**Background:**

It is unknown whether dietary protein consumption can attenuate resistance exercise-induced muscle damage (EIMD). Managing EIMD may accelerate muscle recovery and allow frequent, high-quality exercise to promote muscle adaptations. This systematic review and meta-analysis examined the impact of peri-exercise protein supplementation on resistance EIMD.

**Methods:**

A literature search was conducted on PubMed, SPORTDiscus, and Web of Science up to March 2021 for relevant articles. PEDro criteria were used to assess bias within included studies. A Hedges’ *g* effect size (ES) was calculated for indirect markers of EIMD at  h post-exercise. Weighted ESs were included in a random effects model to determine overall ESs over time.

**Results:**

Twenty-nine studies were included in the systematic review and 40 trials were included in ≥1 meta-analyses (16 total). There were significant overall effects of protein for preserving isometric maximal voluntary contraction (MVC) at 96 h (0.563 [0.232, 0.894]) and isokinetic MVC at 24 h (0.639 [0.116, 1.162]), 48 h (0.447 [0.104, 0.790]), and 72 h (0.569 [0.136, 1.002]). Overall ESs were large in favour of protein for attenuating creatine kinase concentration at 48 h (0.836 [−0.001, 1.673]) and 72 h (1.335 [0.294, 2.376]). Protein supplementation had no effect on muscle soreness compared with the control.

**Conclusion:**

Peri-exercise protein consumption could help maintain maximal strength and lower creatine kinase concentration following resistance exercise but not reduce muscle soreness. Conflicting data may be due to methodological divergencies between studies. Standardised methods and data reporting for EIMD research are needed.

## Introduction

The World Health Organisation physical activity guidelines stipulate adults should perform resistance exercise ≥twice per week to benefit general health, quality of life, and healthy weight maintenance [1]. Resistance training can elicit improved skeletal muscle mass, strength, stability, glucose tolerance, and bone density [2–6]. Nevertheless, unaccustomed resistance exercise, particularly involving eccentric contractions, can damage skeletal muscle fibres [7] mediated by the combined disruption to both sarcomeres and the excitation-contraction coupling system [8, 9]. Resistance exercise-induced muscle damage (EIMD) presents physiological and mechanical consequences that may delay exercise recovery and limit future exercise quality, owing to reduced muscle function. For example, EIMD can induce muscle soreness and swelling and reduce muscle force generating capacity by 50% [10–12], which may persist for seven days post-exercise [13, 14]. Subsequently, acute EIMD can dampen chronic adaptations to resistance training [15]. Successive exposures to comparable exercise stimuli attenuate EIMD owing to the repeated bout effect (RBE) [16–18]. However, even mild symptoms of muscle soreness and weakness could diminish personal motivation to exercise and reduce the frequency and/or quality of exercise sessions. Furthermore, in line with resistance training guidelines, individuals frequently alter exercise load, repetition range, or volume, thus imposing new exercise stimuli and susceptibility to EIMD. Therefore, EIMD may obstruct the benefits of regular (≥twice weekly) and progressive resistance exercise.

EIMD severity can be assessed directly (i.e., through muscle biopsy sampling and magnetic resonance imaging) or indirectly (i.e., through tests of muscle function, subjective soreness, and blood analysis of intramuscular proteins). While direct assessments might seem the preferred option, muscle biopsy sampling is invasive and presents two inherent assumptions: that damage is inflicted by the intended intervention and not the biopsy procedure itself; and that the damage measured within the sample reflects the whole muscle [19, 20]. To this end, indirect markers are preferentially employed to indicate EIMD [21], with isometric and isokinetic tests of muscle function considered the most valid and reliable [22]. Other indirect markers of EIMD, including muscle soreness and blood creatine kinase concentration ([CK]) are limited by their high inter- and intra- individual variability [23, 24] though are frequently assessed in research, allowing for between-study comparisons.

EIMD potentially hinders training adaptations [15] and hence several strategies have been investigated to mitigate EIMD including cryotherapy, massage, stretching, compression garments, electrostimulation [25, 26], and dietary manipulation. Dietary strategies have received considerable recent attention, especially regarding supplemental protein- and amino acid- based products provided peri-exercise [27–33].

Peri-exercise protein consumption is a common strategy to enhance post-exercise recovery and training adaptation, by stimulating increased rates of muscle protein synthesis (MPS) [34]. MPS is stimulated following exercise to repair damaged muscle proteins and MPS rates are further augmented by peri-exercise protein consumption [35]. Given that peri-exercise protein consumption also may reduce muscle damage [27, 28, 33], it follows that protein supplementation may co-benefit muscle recovery by reducing EIMD and enhancing MPS rates. Accordingly, several sources of protein including whey, casein, soy, wheat, and milk have been investigated as nutritional strategies for mitigating EIMD.

Despite extensive research, the evidence for peri-exercise protein supplementation attenuating EIMD remains inconclusive. Following resistance exercise, declines in maximal strength have been attenuated with milk protein ingestion in some [36, 37], but not all [38–40] studies. Recently, regular consumption of whey but not pea protein during 96 h of exercise recovery lowered peak elevations in serum [CK] relative to water ingestion [41]. However, whey protein intake failed to ameliorate muscle soreness; consistent with other observations, irrespective of feeding timing [42, 43]. Moreover, the impact of whey protein supplementation on EIMD is apparently influenced by whether hydrolysed or isolated whey protein is provided, despite equivalent dosing protocols [44, 45]. The variety of exercise protocols, protein dosing and timing regimes, participant characteristics, dietary controls, and measurement tools employed among studies likely contribute to the diverse findings. Drawing conclusions on the efficacy of dietary protein for managing EIMD requires a systematic approach with account for methodological design.

The systematic reviewing of relevant literature has failed to produce definitive conclusions, perhaps due to either overly broad or narrow inclusion criteria [27, 31]. No review yet has explicitly analysed studies whereby a variety of protein supplements were consumed in conjunction with resistance exercise. Pasiakos and colleagues [31] systematically reviewed studies that utilised varied exercise protocols (resistance and endurance) and provided protein- or amino acid- based supplements. Resistance exercise typically causes more severe EIMD than endurance exercise, although only five of the 27 included studies involved resistance exercise alongside protein consumption [31]. Therefore, this review cannot conclude the impact of protein supplementation on resistance EIMD. Conversely, a systematic review (*n* = 8) by Davies and colleagues [27] assessed the impact of protein supplementation on EIMD following resistance exercise. However, the inclusion criteria were limited to studies exclusively examining the response of muscle function to whey protein supplementation, without consideration for other protein sources and EIMD markers. The accompanying meta-analysis revealed an overall small-medium beneficial effect of whey protein in restoring muscle function during exercise recovery. Nonetheless, the impact of a range of protein sources on various EIMD markers is currently unknown. Therefore, this systematic review and meta-analysis focused on studies examining the impact of peri-exercise protein supplementation on indirect markers of muscle damage following acute resistance exercise. This review will inform protein intake recommendations specifically for resistance exercise recovery.

## Methods

### Inclusion criteria

The analysis was confined to studies published in English-language journals that met the following criteria: (1) an experimental trial involving acute (single-bout) resistance exercise; (2) includes ≥one indirect measure of muscle damage; (3) muscle damage is measured for ≥24 h post-exercise; (4) includes peri-exercise supplementation with protein versus control (including carbohydrate); (5) involves adult participants with no known clinical condition or musculoskeletal injury. Studies were excluded if conducted in participants <18 y, animals, or in vitro models, or included another protein supplement as the control, supplements containing therapeutic or ergogenic aids (e.g., polyphenols, antioxidants), or physiotherapeutic/pharmaceutical methods targeting muscle damage (e.g., massage, cryotherapy). Studies involving endurance-type exercise, unloaded resistance exercise (e.g., drop-jumps), concurrent exercise (if the dominant exercise was not resistance-type), or chronic resistance training were excluded, as these exercise modes induce varied severities of EIMD. Therefore, it would be inappropriate for this meta-analysis to group different modes of exercise, and further, this review aimed to inform sport nutrition guidelines exclusively for resistance exercise.

### Search strategy

The literature search was conducted according to the Preferred Reporting Items for Systematic Reviews and Meta-Analyses (PRISMA) guidelines [46] on PubMed, SPORTDiscus, and Web of Science databases for studies published up to March 2021 using the following syntax: (muscle damage OR muscle injury OR muscle soreness OR exercise recovery OR exercise-induced muscle damage OR EIMD) AND (protein OR “milk*” OR whey OR soy OR casein OR wheat OR beef) NOT (running OR cycling OR rats OR mice). Articles were assessed for eligibility by two independent reviewers using the Rayyan software [47]. The reference lists of eligible articles were also screened.

### Coding of studies and data extraction

Studies were read and individually coded by two independent reviewers (AP and LM) for the following variables: (1) author, title, and year of publication: (2) participant demographic (sex, age (categorised as per Schoenfeld et al. [48]) training status, and sample size); (3) resistance exercise protocol, including exercise mode, load, set and repetition number, and inter-set rest duration; (4) type of protein and control supplement used; (5) total daily protein intake; (6) protein dose and timing protocol; (7) assessment methods of muscle damage; (8) measurement time-points in relation to exercise; and (9) significant findings. For analyses, mean and standard deviation (SD) (absolute or change from baseline), and sample size data were extracted for each variable and time-point for treatment and control groups. Study authors were contacted to provide raw data and if not received, these data were extracted from reported figures using WebPlotDigitizer.

### Methodological quality

Study quality was assessed by two independent reviewers (AP and LM) based on the 11-point Physiotherapy Evidence Database (PEDro) scale, which is considered reliable and valid for quality-assessing randomised controlled trials [49, 50]. Ratings were categorised as: 0–3 = ‘poor’; 4–5 = ‘fair’; 6–8 ‘good’; and 9–10 = ‘excellent’.

### Meta-analyses

Each within-study comparator group (protein vs control) was treated as an independent trial. A meta-analysis was conducted if the total number of trials (*k*) was ≥3 to generate *k* independent effect sizes (ESs). Separate meta-analyses (16 total) were conducted at each post-exercise time-point (<24, 24, 48, 72, and 96 h) for the most measured variables: isometric MVC, isokinetic MVC, serum/plasma [CK], and muscle soreness visual analogue/rating scale (VAS/VRS) score. If multiple measures of these variables were obtained, e.g., leg extension and flexion MVC or active and passive muscle soreness, the mean of these values was used in the analysis.

For each trial, a Hedges’ *g* ES with correction for small positive bias was calculated due to dissimilar within-study group sample sizes [51]. For MVC and [CK], ESs were calculated from mean and SD values as a percent change from baseline. For muscle soreness, there were insufficient data to use percent change from baselines values, due to muscle soreness being either not measured or reported as 0 at baseline. Therefore, muscle soreness ESs were calculated using the absolute mean and SD at each time-point. Weighted ESs were calculated using the standard error of the effect and adjusted with Tau squared (*Ʈ²)*.

Trial heterogeneity within each meta-analysis was assessed using Cochran’s Q (*Q*) and *I*^*2*^ [52]. Due to the moderate-substantial heterogeneity detected, a random-effect model was used to calculate pooled ESs, which are reported with 95% confidence intervals [51]. ESs are interpreted as 0.2 = ‘small’; 0.5 = ‘medium’; and 0.8 = ‘large’ with statistical significance determined by zero overlap of the 95% confidence interval range [53]. To identify potentially influential trials, a sensitivity analysis was conducted by performing meta-analyses with removal of each trial one at a time. Trials were considered influential if their removal resulted in the pooled ES changing from significant to non-significant, or vice versa. Pooled ESs with removal of influential trials are reported in the manuscript text and forest plots display all trials.

The magnitude of EIMD was determined for trials included in the meta-analyses based on the relative peak reduction from baseline in MVC as per Paulsen et al. [54]: mild = <20%, moderate = 20–50%, and severe = >50% reduction. For studies providing insufficient data to be meta-analysed (i.e., do not report mean change and variation), or when *k* < 3, the mean percent change values were calculated.

## Results

### Study quality and overview

**Fig. 1:**
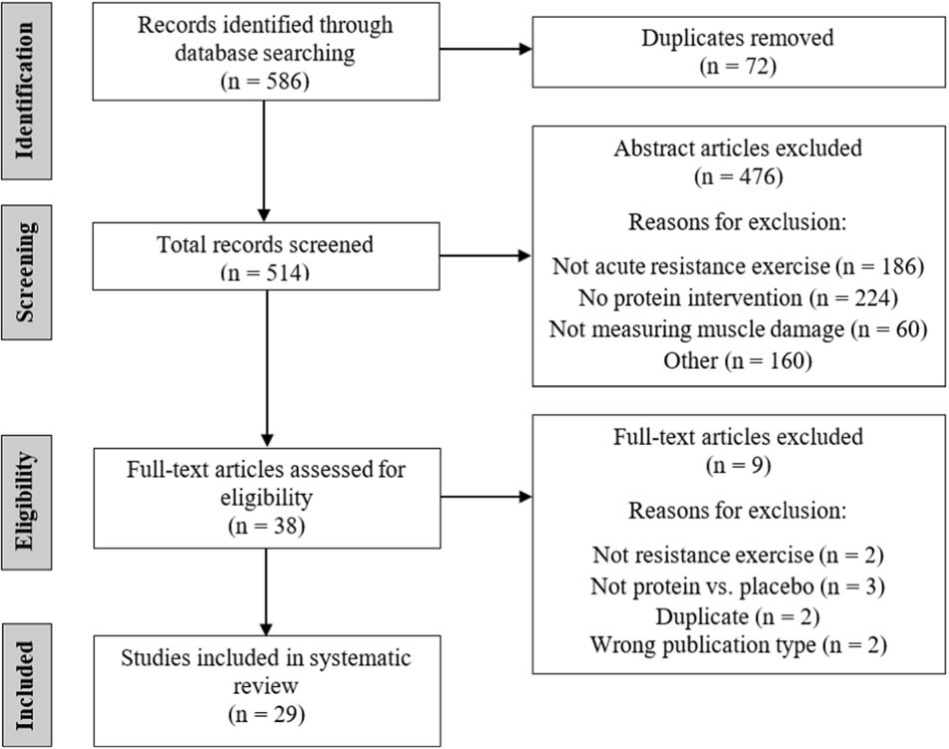
PRISMA flow diagram. Flow diagram of the literature search process.

The literature search yielded 586 articles, of which 38 potentially met the inclusion criteria based on abstract screening (Fig. 1). After full-text screening, 29 studies were confirmed to meet the inclusion criteria and were included in the systematic review (Table 1).

**Table 1:** Summary of studies included in the review (*N* = 29).

Reference (Quality rating)	Study design Participant characteristics	Mode(s) of resistance exercise; load; sets × reps (rest period duration)	Type of protein/placebo provided [Isoenergetic (I), Non-Isoenergetic (NI)]	Protein dose and timing protocol	Daily protein intake (g kgBM^−1^) excluding supplement	Daily protein intake (g) including supplement	Markers of muscle damage	Time points of measures around exercise bout	Primary findings
Baty et al. [55] (9; excellent)	Parallel *N* = 34 M; young; untrained	High pull, latissimus pull-down, overhead press, bench press, leg extension, leg curl, leg press; 8RM; 3 × 8 (100 s)	Protein + CHO (1.5 + 6.2%-1.5%) Control (NI; electrolyte)	355 mL −30 min pre-Ex, 177 mL Pre & during-Ex, 355 mL Post-Ex	NR	NR	Plasma [CK and Mb]	−30 min Pre During Post +1 h +6 h +24 h	↑ [Mb] at +6 h and [CK] at +24 h in control
Bird et al. [86] (8; good)	Crossover *N* = 15 M; young; trained	Back squat, deadlift, leg press, leg extension, leg curl; 8–15 RM; 4 × 8–15 (90 s)	Triphasic protein + CHO Control (NI; artificial sweetener)	8 g + 5.5 g Pre-Ex 6 g + 20 g During-Ex 22 g + 20 g Post-Ex	1.5 1.5	165 129	Serum [CK and CRP] Muscle soreness VAS (with pressure algometry) and VRS (bodyweight squats) CMJ peak power	Pre During Post +30 min +24 h	↑ [CK] AUC from pre to +30 min in control
Buckley et al. [44] (9; excellent)	Parallel *N* = 28 M; young; untrained	Eccentric unilateral knee extensions; maximal; 100	Whey protein hydrolysate _(a)_ Whey protein isolate _(b)_ Control (NI; flavoured water)	25 g 25 g 250 mL 0, 6 & 22 h post-Ex	NR	NR	Serum [CK] Plasma [TNF-α] Muscle soreness VAS (extended leg with 5 kg suspension) Isometric MVC knee extensor	Pre Post +1 h +2 h +6 h +24 h	↑ Isometric MVC at +6–24 h in whey protein hydrolysate compared to isolate and control
Burnley et al. [57] (9; excellent)	Crossover *N* = 21 M; young; untrained	Eccentric knee extensions; 100% concentric 1RM; 10 × 10 (1 min)	Whey protein CHO (I; sugar) Control (NI; artificial sweetener)	0.4 g kgBM^−1^ 0.4 g•kgBM^−1^ 240 mL Post-Ex	0.9 1.0 1.0	107 83 78	Plasma [CK] Muscle soreness VAS (passive) Isometric MVC knee extensor and flexor Knee extensor and flexor mean power	Pre Post +24 h +48 h	↔
Cockburn et al. [36] (6; good)	Parallel *N* = 24 M; young; trained	Unilateral knee flexions; maximal; 6 × 10 (90 s)	Milk-based Protein + CHO _(a)_ Semi-skimmed milk (NI) _(b)_ CHO (NI; sports drink) Control (NI; water)	17 g 17 g 0 g 0 g All 500 mL 0 & 2 h post-Ex	NR	NR	Serum [CK and Mb] Muscle soreness VAS (passive) Isokinetic MVC knee flexion	Pre +24 h +48 h	↑ Isokinetic MVC and ↓ [CK and Mb] at +48 h in CHO-Protein and Milk compared to CHO
Cockburn et al. [38] (4; fair)	Parallel *N* = 32 M; young; trained	Unilateral knee flexions; maximal; 6 × 10 (90 s)	Milk-based Protein + CHO Control (NI; water)	33 g (1000 mL) Pre-Ex _(a)_ *or* Post-Ex _(b)_ *or* 24 h post-Ex _(c)_	NR	NR	Serum [CK] Muscle soreness VAS (maximal leg flexion) Isokinetic MVC knee flexor Reactive strength index	Pre +24 h +48 h +72 h	No significant interactions between supplement condition and time point on muscle damage markers
Cockburn et al. [39] (3; poor)	Parallel *N* = 24 M; young; trained	Unilateral knee flexions; maximal; 6 × 10 (90 s)	Semi-skimmed milk Control (NI; water)	17 g (500 mL) _(a)_ *or* 34 g (1000 mL) _(b)_ Post-Ex	NR	NR	Serum [CK and Mb] Plasma [IL-6] Muscle soreness VAS (passive and during maximal leg flexion) Isokinetic MVC knee flexor	Pre +24 h +48 h +72 h	No significant interactions between supplement condition and time point on muscle damage markers
Cockburn et al. [64] (5; fair)	Parallel *N* = 14 M; young; trained	Eccentric unilateral knee flexions; 6 × 10 (90 s)	Semi-skimmed milk Control (NI; water)	17 g (500 mL) Post-Ex	NR	NR	Serum [CK and Mb] Muscle soreness VAS (passive and during all performance measures) CMJ height Reactive strength index 15 m sprint Agility time Intermittent shuttle test	Pre Post +24 h +48 h +72 h	Significantly faster 15 m sprint time during shuttle test with milk than placebo
Cooke et al. [91] (9; excellent)	Parallel *N* = 17 M; young; untrained	Unilateral leg press, leg extension, leg curl; 120% 1RM; 4 × 10 (3 min)	Whey protein isolate+CHO CHO (I; glucose)	1.5 g kgBM^−1^ Post-Ex Then 27 g 4 × daily for 14 d	0.9 0.8	177 63	Plasma [CK and LDH] Isometric MVC knee extensor Maximal isokinetic knee extensor and flexor strength	Pre +30 min +1 h +2 h +4 h +24 h +48 h +72 h +96 h +7 d +14 d	↑ Isometric MVC at +72 h and +7 d with whey protein
Dale et al. [45] (9; excellent)	Parallel *N* = 39 M; young; untrained	Eccentric knee extension; maximal; 100	Whey protein hydrolysate (one of three forms) _(a)_ Whey protein isolate (I) _(a)_ Control (NI; flavoured water)	25 g Post-Ex then once daily 25 g 250 mL	NR	NR	Isometric MVC knee extensor	Pre +1 h +2 h +6 h +24 h +48 h +72 h +7 d	↔
Draganidis et al. [37] (10; excellent)	Crossover *N* = 11 M; young; trained	Eccentric unilateral knee extensions; 20 × 15 (30 s)	Milk protein concentrate CHO (I; maltodextrin)	20 g 0, 3, 6 & 9 h Post-Ex Daily for 8 d 0 g	2.1 1.0	159 79	Serum [CK] Muscle soreness VAS (palpation after squats) Isometric MVC knee extensor	Pre +6 h +24 h +48 h +72 h +96 h +5 d +6 d +7 d +8 d	↑ Isometric MVC and lower muscle soreness at +1–5 d with milk protein, ↓ [CK] at +24–48 h with milk protein
Farup et al. [13] (9; excellent)	Parallel *N* = 24 M; young; untrained	Eccentric unilateral knee extension; maximal; 15 × 10 (1 min)	Whey protein hydrolysate+ CHO CHO (I)	28 g + 28 g 0, 3 & 6 h Post-Ex 3× daily for 2 d 56 g	1.2 1.2	170 86	Serum [CK] Muscle soreness VAS (during single-leg squat) Isometric MVC knee extensor	Pre +24 h +48 h +72 h +96 h +7 d	↑ Muscle soreness at +96 h in whey protein
Gee et al. [70] (10; excellent)	Parallel *N* = 30 M; young; trained	Back squat, bench press, deadlift, military press, bench pull; 75% 1RM; 4 × 8 (2 min)	Whey protein hydrolysate + CHO _(a)_ Flavoured milk (I) _(b)_ CHO (I)	33 g + 98 g 33 g + 98 g 133 g Post-Ex	NR	NR	Muscle soreness VAS Isokinetic MVC knee extensor and flexor CMJ height Medicine-ball throw distance	Pre +24 h +48 h	↔
Grubic et al. [61] (7; good)	Crossover *N* = 12 M; young; trained	Bench press, incline bench press, back squat, leg press, lunges, leg extension, latissimus pulldown, row, bicep curls, triceps pulldown, preacher curls; 70% 1RM; 3 × 10 (2 min) Followed by sprint intervals	Whey protein + CHO food bar CHO (NI; dextrose gel)	20 g + 25 g 25 g Pre-, during, & Post-Ex	1.6 1.6	146 130	Serum [CK, IL-8 and TNF-α] Muscle soreness VAS (with pressure algometry) Isokinetic MVC knee extensor and flexor	Pre During Post +48 h	↔
Hasegawa et al. [58] (4; fair)	Crossover *N* = 6 M; young; untrained	Leg extension, leg press, row, chest fly; 80% 1RM; 3 × 10 (1 min)	Egg white protein _(a)_ Soy protein (I) _(b)_ Control (NI; water)	20 g 20 g Pre-Ex	0.9 0.8 0.8	76 70 50	Serum [CK] Muscle soreness VAS (passive)	Pre Post +30 min +24 h +48 h +72 h	↔
Hirose et al. [84] (6; good)	Crossover *N* = 6 M; young; untrained	Calf-raise; 100% BM; 5 × 15 (60 s)	Milk peptide Control (NI; no supplement)	5 g Pre- & Post- Ex Twice daily for 5 d	NR	NR	Plasma [CK] Muscle soreness VAS (palpation and unloaded extension)	Pre Post +12 h +24 h +48 h +72 h +96 h +5 d +8 d	↓ [CK] at +72 h and +5 d with milk peptide, ↓ Muscle soreness with milk peptide
Hoffman et al. [59] (8; good)	Parallel *N* = 15 M; young; trained	Back squat, deadlift, barbell lunge; 80% 1RM; 4 × 10 (90 s)	Protein blend (collagen, whey, and casein isolates, plus 250 mg BCAAs) CHO (NI; maltodextrin)	42 g Pre- & Post- Ex for 3 d	2.0 2.0	277 193	Serum [CK] Muscle soreness VRS Squat peak power	Pre Post +24 h +48 h	↔
Ives et al. [92] (7; good)	Parallel *N* = 60 M; young; untrained	Eccentric unilateral knee extension; maximal; 100	Whey protein hydrolysate CHO (I; sugar flavoured water)	31 g 0, 6 & 22 h Post-Ex 250 mL	1.3 1.4	189 96	Muscle soreness VAS (extended leg with and without 5 kg suspension) Isometric MVC knee extensor Isokinetic MVC knee extensor Thigh circumference	Pre Post +1 h +2 h +6 h +24 h	↑ Peak isokinetic MVC at +24 h with protein compared to CHO
Karakus and Akkurt [83] (5; fair)	Parallel *N* = 24 M; young; trained	Bench press, chest fly, reverse fly, shoulder press, triceps pushdown, bicep curl, back squat, leg press, leg extension, leg flexion, adductor, calf press; 80%, 90%, and 100% 1RM; 3 × 10	Whey protein Control (NI; no supplement)	35 g Post-Ex then daily	1.0 1.0	105 70	Serum [CK and Mb] Muscle soreness VAS	Pre Post +24 h +48 h +72 h	↑ Muscle soreness at +24–72 h with protein
Kim et al. [42] (7; good)	Parallel *N* = 32 M; young; untrained	Eccentric elbow flexions; 2 × 25	Whey protein Control (NI; no supplement)	1.5 g kgBM^−1^ Pre-Ex _(a)_ *or* Post-Ex _(b)_ *or* Pre- & Post-Ex _(b)_	NR	NR	Serum [CK] Muscle soreness VAS Isometric MVC elbow flexor ROM elbow flexor	Pre Post +24 h +48 h +72 h +96 h	↔
Naclerio et al. [87] (9; excellent)	Crossover *N* = 10 M; young; trained	Bench press, back squat, row, lunge, deadlift, box step-up, hang clean; 40–60% 1RM; 3 × 15 (30 s) 3 consecutive days	Protein blend (whey isolate, beef hydrolysate, glutamine + CHO) CHO (I; maltodextrin)	18 g + 38 g Post-Ex for 3 d 55 g	1.7 1.7	145 127	Muscle soreness VAS (bodyweight squat) Bench press 1RM and maximal power CMJ height	Pre +1 h +24 h +48 h	↑ CMJ height at +1 h, maximal strength at +24 h, and maximal power at +24–48 h with protein
Nieman et al. [41] (10; excellent)	Parallel *N* = 92 M; young; untrained	Bench press, eccentric latissimus pulldown, eccentric leg extension, eccentric leg curl, split squats, and various unloaded exercises; ≤maximal; 2–4 × 3–15 (30–60 s)	Whey protein isolate _(a)_ Pea protein isolate (I) _(b)_ Control (NI; water)	0.3 g kgBM^−1^ 0.3 g kgBM^−1^ 237 mL Pre- & Post- Ex and pre-sleep for 4 d	NR	NR	Serum [CK, Mb, LDH and CRP] Muscle soreness VAS Isometric MVC deadlift dynamometer Bench press repetitions CMJ height 30-s Wingate	Pre Post +24 h +48 h +72 h +96 h	↓ [CK] at +72–96 h and [Mb] at +48–96 h with whey protein compared to water, ↔ between pea protein and water
Philpott et al. [63] (7; good)	Parallel *N* = 36 M; young; trained	Eccentric unilateral knee flexion; 12 sets (each leg) (1 min)	Whey protein + Leucine + CHO CHO (NI)	15 + 1.8 + 20 g Twice daily for 6 wk pre-Ex 24 g	NR	NR	Serum [CK] Plasma [CRP] Muscle soreness VAS (passive and unloaded leg extension) Isokinetic MVC knee flexor Yo-Yo intermittent endurance test Soccer passing skills test	Pre +24 h +48 h +72 h	↔
Rankin et al. [40] (7; good)	Parallel *N* = 32 (16 M, 16 F); young; trained	Eccentric knee flexion; maximal; 6 × 10 (90 s)	Milk CHO (I)	17 g (500 mL) Post-Ex 500 mL	NR	NR	Serum [CK] Muscle soreness VAS (passive and maximal leg flexion) Isokinetic MVC knee flexor CMJ height 20 m sprint	Pre +24 h +48 h +72 h	No significant interactions between supplement condition and time point on muscle damage markers
Samadi et al. [56] (6; good)	Parallel *N* = 28 M; young; untrained	Leg press, leg curl, leg extension, shoulder press, latissimus pulldown, bench press, bicep curl; 70–75% 1RM; 3 × 8 (1 min)	9% whey protein + CHO in various ratios: 1:4 1:3 1:2 Control (NI; artificial sweetener)	24 g divided into 4 doses 2.5 mL•kgBM^−1^ solution Pre-Ex and 3 doses during Ex at 15-min intervals	NR	NR	Plasma [CK and Mb] Muscle soreness VRS (passive)	Pre +1 h +24 h +48 h	↑ [CK and Mb] at +24 h in control
Saracino et al. [60] (9; excellent)	Parallel *N* = 32 M; middle-aged; untrained	Eccentric knee extensions and flexions; maximal; 5 × 15 (2 min)	Whey protein hydrolysate _(a)_ Whey protein isolate _(b)_ Rice and pea protein _(c)_ Control (NI)	40 g Pre-sleep before Ex and for 2 d post-Ex and 25 g bolus post-Ex	1.1 1.1 1.1 1.1	155 156 151 84	Plasma [CK, IL-6 and IL-10] Muscle soreness VAS (unloaded leg extension) Isometric MVC knee extensor and flexor Isokinetic MVC knee extensor and flexor Thigh circumference	Pre +24 h +48 h +72 h	↔
West et al. [62] (9; excellent)	Crossover *N* = 12 M; young; trained	Bench press, latissimus pulldown, barbell shoulder press, row, leg press, leg extension; 75% 1RM; 4 × 10 (2 min) Or non-exercising control	Whey protein CHO (I) Control (No supplement)	25 g 0 & 10 h Post-Ex	NR	NR	Isometric MVC knee extensor CMJ performance Knee extensor repetitions to failure at 75% 1RM 30 s Wingate	Pre Post +10 h +24 h	Small-to-moderate beneficial effects on isometric MVC, anaerobic power, and CMJ at +10 h and moderate beneficial effects on isometric MVC, anaerobic power and muscular endurance at +24 h with protein
White et al. [43] (9; excellent)	Parallel *N* = 27 M; young; untrained	Eccentric unilateral knee extensions; maximal; 5 × 10 (1 min)	Whey protein + CHO Control (NI; artificial sweetener)	23 + 75 g Pre-Ex _(a)_ *or* Post-Ex _(b)_ Pre- & Post- Ex	NR	NR	Serum [CK] Muscle soreness VAS Isometric MVC knee extensor	Pre +6 h +24 h +48 h +72 h +96 h	↔
Wojcik et al. [93] (6; good)	Parallel *N* = 26 M; young; untrained	Eccentric knee extension; 120% of concentric 1RM; 10 × 10 (1 min)	Flavoured milk protein + CHO CHO (I; Gatorade) Control (NI; artificial sweetener)	25 + 70 g 0 and 2 h post-Ex 95 g 0 g	1.5 1.5 1.5	164 113 113	Serum [CK, IL-1, IL-6 and TNF-α] Muscle soreness VRS (passive) Isometric MVC knee extensor	Pre Post +3 h +6 h +24 h +48 h +72 h +96 h +5 d +6 d	↔

Arrows indicate a significant difference between groups (↑ = higher, ↓ = lower, ↔ = no significant differences); methodological quality rating categorised as 0–3 = ‘poor’, 4–5 = ‘fair’, 6–8 ‘good’, and 9–10 = ‘excellent’; participant age is classified as young (18–39 y), middle-aged (40–64 y), and older adults (≥ 65 y) [48]; subscripts (a), (b), and (c) denote separate trials within a study; *M* males, *F* females, *1RM* one repetition maximum, *CHO* carbohydrate, *BCAAs* branched chain amino acids, *Ex* exercise, *NR* not reported, *CK* creatine kinase, *Mb* myoglobin, *LDH* lactate dehydrogenase, *IL* interleukin, *TNF-α* tumor necrosis factor-alpha, *CRP* C-reactive protein, *VAS* visual analogue scale, *VRS* visual rating scale, *CMJ* counter movement jump, *MVC* maximal voluntary contraction, *ROM* range of motion, *PPT* pressure pain threshold.

The 29 studies consisted of 45 trials, of which 26 studies and 40 trials were included in ≥1 meta-analyses. Three studies were not included in any meta-analysis [55–57], due to insufficient data and the mean percent change values are reported in Table 2. Methodological quality ratings are included in Table 1. The mean and median rating of study quality were 7 and 8, respectively, indicative of good quality. Only one study was categorised as poor and 13 as excellent.

**Table 2:** Mean percent change values from trials not included in the meta-analyses.

		Mean percent change (range)				
Variable	Group	Pre-Post	Pre-24 h	Pre-48 h	Pre-72 h	Pre-96 h
Isometric MVC	Protein	−29.9–−2.4	−31.9–0.0	−32.3–−0.7	−27.3–2.4	−23.0–6.1
	Control	−36.7–−2.6	−38.0–−3.2	−32.1–−3.8	−32.6–−2.6	−22.2–3.8
Isokinetic MVC	Protein	−24.2–−1.5	−26.2–0.6	−42.1–−3.2	–	–
	Control	−20.0–0.7	−25.5–−8.8	−33.7–−2.1	–	–
CK	Protein	2.0–166	29.8–1660.2	15.9–7532.3	152.0–18769.1	166.6–33222.0
	Control	−7.9–283.7	37.1–1605.7	42.7–3770.8	363.8–10471.4	325.3–13907.6

Time is in relation to the exercise bout; *MVC* maximal voluntary contraction, *CK* creatine kinase

In total, 763 participants (94% young males) were included. Fourteen studies were conducted with trained individuals and 15 with untrained. The muscle-damaging resistance exercise was restricted to lower-body muscle groups in most studies; upper-body in one study [42], and whole-body in 9 studies. Muscle contractions were concentric-eccentric (*n* = 16 studies) or eccentric-only (*n* = 13 studies). The magnitude of EIMD was predominantly mild or moderate, with only one study reporting severe EIMD [42]. EIMD magnitude seemingly did not influence the response to protein supplementation (Supplementary Table S1).

Protein was provided pre-exercise (*n* = 1) [58], post-exercise (n = 16 studies), or pre- and post- exercise (*n* = 12), including 3 studies that investigated supplementation timing [38, 42, 43]. Whey protein (including hydrolysed and isolated forms) was the most common protein source used, either alone (*n* = 10 studies) or combined with carbohydrate (*n* = 10 studies). Eight studies provided milk-based protein and 4 studies included other protein sources (whey, casein, and collagen blend [59], pea protein [41], rice and pea protein [60], egg white and soy protein [58]). The control group supplements were either carbohydrate-based (*n* = 9), a non-isoenergetic liquid (e.g., artificially-sweetened water) (*n* = 11), both (*n* = 5), or no supplement provided (*n* = 3). Except for one study [61], all supplements were liquid. Daily protein intake with exclusion of the supplement was adequate in all trials in the protein groups (0.8–2.1 g kgBM^−1^) and the control groups (0.8–2.0 g kgBM^−1^). With inclusion of the supplement, absolute daily protein intake ranged 70–277 and 50–193 g in protein and control groups, respectively with a mean between-group difference of 56 g. Sixteen studies did not report daily nutrient intake.

### Isometric maximal voluntary contraction

**Fig. 2:**
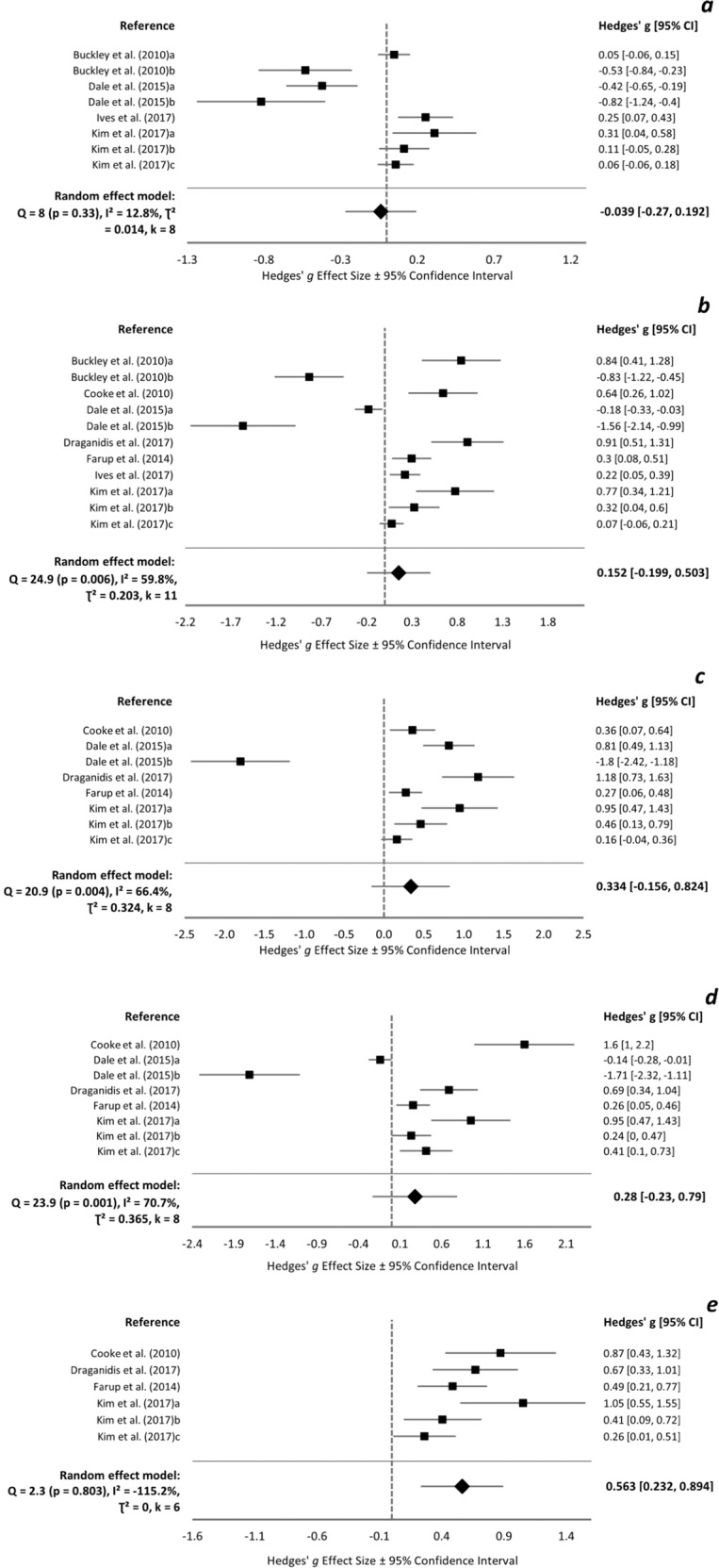
Isometric MVC forest plot. Forest plot of Hedges’ g effect sizes with 95% confidence intervals for the effect of protein supplementation compared to control on the change from baseline in isometric maximal voluntary contraction at **a** <24 h post-exercise, **b** 24 h post-exercise, **c** 48 h post-exercise, **d** 72 h post-exercise, and **e** 96 h post-exercise. A positive effect size indicates a beneficial effect of protein supplementation compared to control. All eligible trials, including outliers, are presented and included in the analysis.

Baseline isometric MVC ranged 133.7–292.4 and 125.3–314.0 Nm in protein and control groups, respectively. Three trials did not report baseline data [13, 42, 62]. Meta-analyses for isometric MVC change from baseline between protein and control groups were conducted at <24 h (*k* = 8), 24 h (*k* = 11), 48 h (*k* = 8), 72 h (*k* = 8), and 96 h (*k* = 6). Overall ESs were small and insignificant at <24–72 h (Fig. 2a–d). An influential trial was detected at 48 h [45]_b_ and its removal resulted in significant medium positive effects of protein (pooled ES = 0.564 [0.049, 1.080]). There was an overall beneficial effect of protein at 96 h (ES = 0.563 [0.232, 0.894]) (Fig. 2e).

### Isokinetic maximal voluntary contraction

**Fig. 3:**
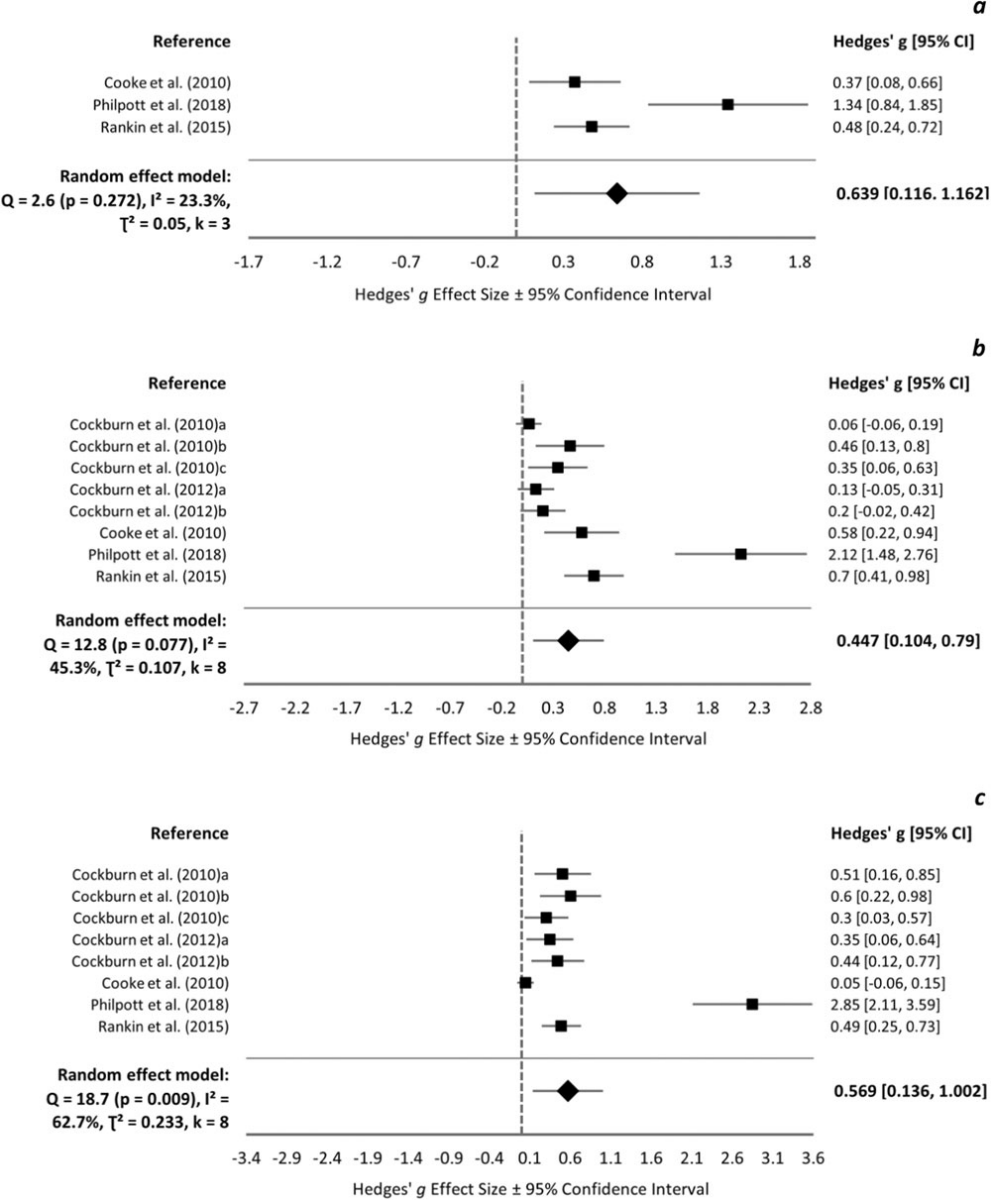
Isokinetic MVC forest plot. Forest plot of Hedges’ g effect sizes with 95% confidence intervals for the effect of protein supplementation compared to control on the change from baseline in isokinetic maximal voluntary contraction at **a** 24 h post-exercise, **b** 48 h post-exercise, and **c** 72 h post-exercise. A positive effect size indicates a beneficial effect of protein supplementation compared to control.

Isokinetic MVC at baseline ranged 74.5–188.0 and 72.1–183.0 Nm in protein and control groups, respectively, and was not reported in 2 studies [38, 63]. Meta-analyses were conducted at 24 h (*k* = 3), 48 h (*k* = 8), and 72 h (*k* = 8). Overall ESs were small-medium in favour of protein and reached statistical significance at all time-points (Fig. 3a–c). Philpott et al. [63] was identified as influential and its removal resulted in insignificant overall ESs at 48 (0.319 [−0.036, 0.675]) and 72 h (0.371 [−0.08, 0.822]). There was no clear impact of protein type, time or duration of supplementation, muscle group exercised, contraction type, or the training status of participants on the change in isometric and isokinetic MVC at 24 h (supplementary Fig. S1a).

### Creatine kinase

Baseline [CK] ranged 33.7–307.0 and 43.5–540.5 IU L^−1^ in protein and control groups, respectively. There were insufficient data to meta-analyse trials at <24, 24, and 96 h. At 48 h, 6 trials produced significant medium-large positive effects of protein and the overall effect was borderline significant (ES = 0.836 [−0.001, 1.673]). One influential trial [64] produced large negative effects (−1.88 [−2.59, −1.16]) and, once removed, the overall effect became large in favour of protein (1.252 [0.354, 2.151]). There were large positive effects of protein at 72 h (overall ES = 1.335 [0.294, 2.376]). Removal of one influential trial [40] resulted in insignificant overall effects (0.952 [−0.170, 2.075]).

### Muscle soreness

**Fig. 4:**
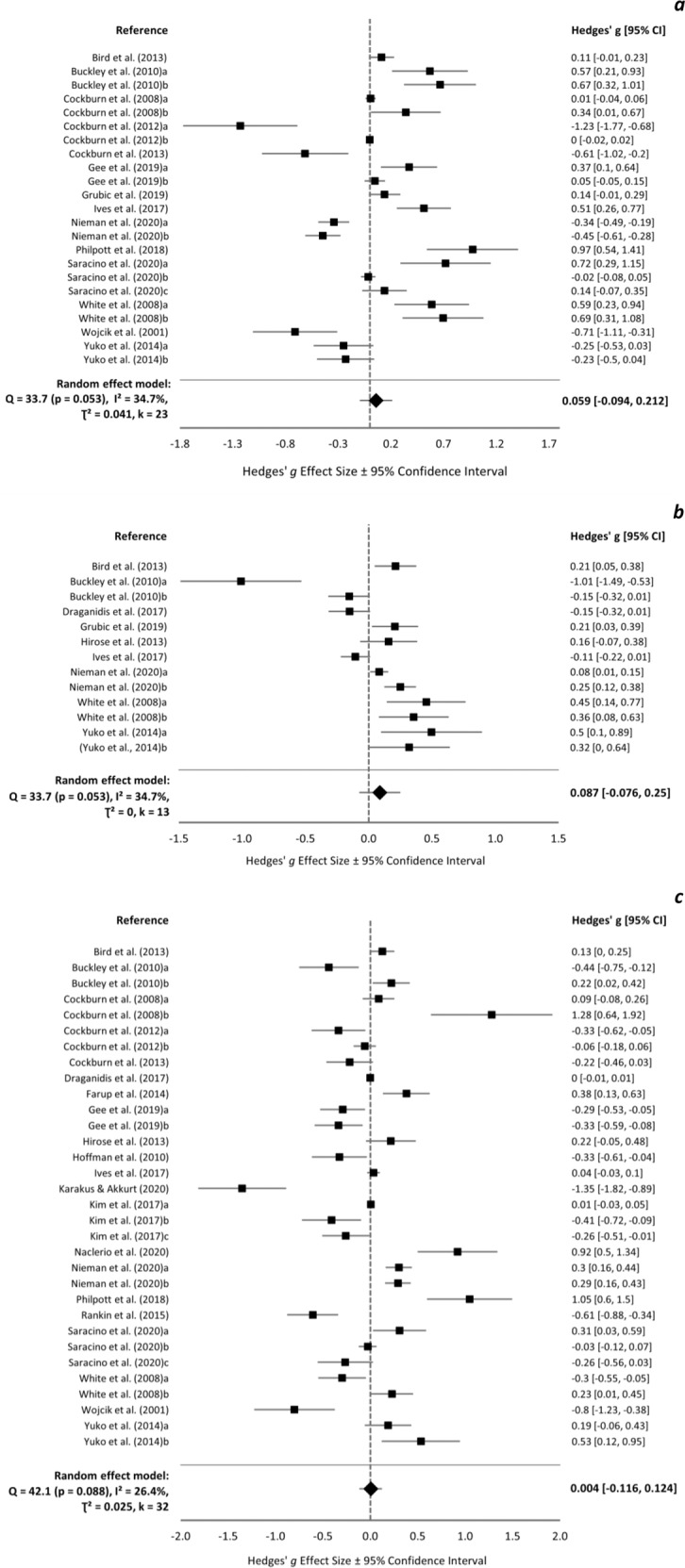

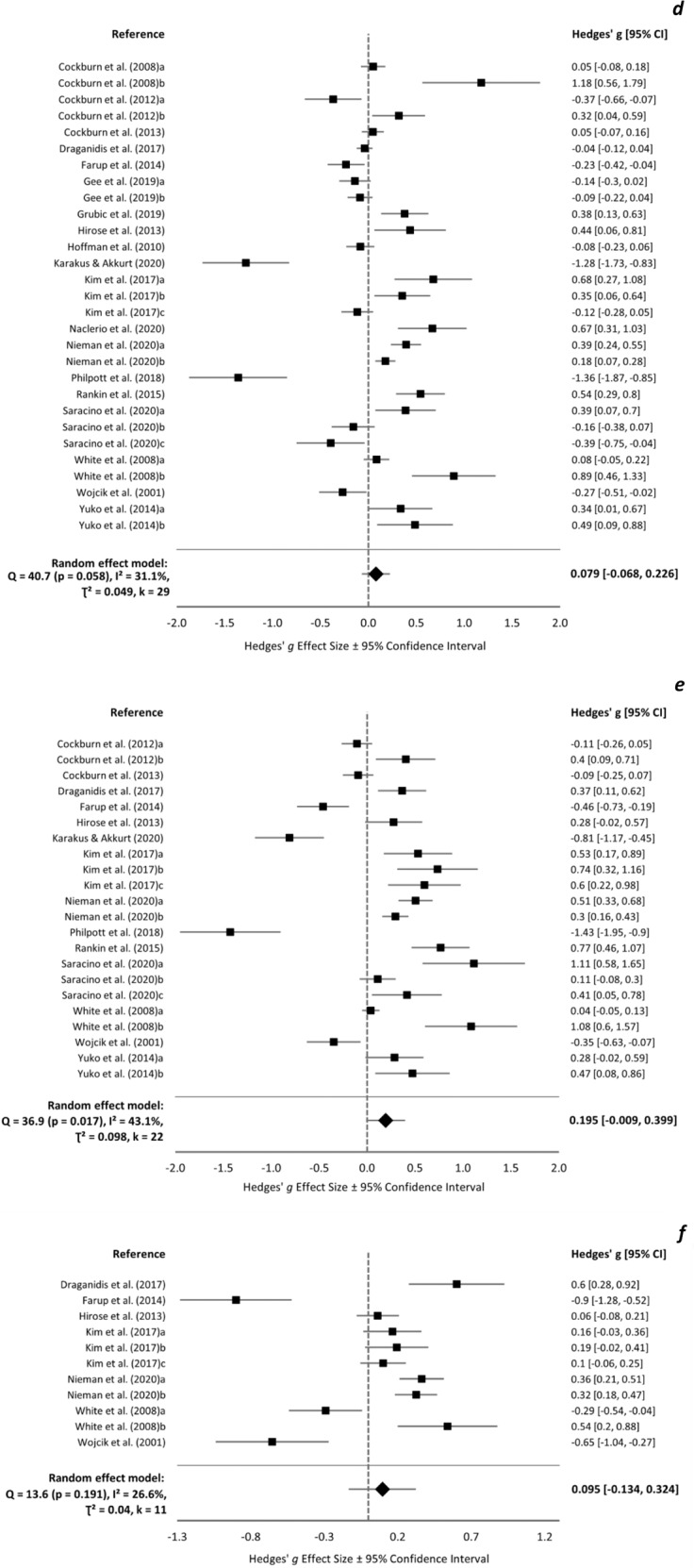
Muscle soreness forest plot. Forest plot of Hedges’ g effect sizes with 95% confidence intervals for the effect of protein supplementation compared to control on muscle soreness ratings at **a** pre-exercise, **b** <24 h post-exercise, **c** 24 h post-exercise, **d** 48 h post-exercise, **e** 72 h post-exercise, and **f** 96 h post-exercise. A positive effect size indicates a beneficial effect of protein supplementation compared to control. All eligible trials, including outliers, are presented and included in the analysis.

Baseline muscle soreness ranged 0.0–27.9 and −0.5–31.4 mm in protein and control groups, respectively, and was not reported in one study [38]. Meta-analyses were conducted at baseline (*k* = 23), <24 h (*k* = 13), 24 h (*k* = 32), 48 h (*k* = 29), 72 h (*k* = 22), and 96 h (*k* = 11). There was no overall effect of supplement group on muscle soreness at any time-point (Fig. 4a–f). At 72 h, one influential trial was identified [63] and, upon removal, a significant positive effect of protein was found (overall ES = 0.230 [0.054, 0.406]). Protein supplementation appears more beneficial for muscle soreness in untrained individuals, following concentric exercise, and with a single day of supplementation (supplementary Fig. S1b).

## Discussion

Peri-exercise protein consumption has beneficial effects on preserving acute muscle strength and blunting [CK] following muscle damaging resistance exercise in young males. Reductions in isokinetic MVC were significantly attenuated by protein in all 8 trials at ≥1 time-point, with no negative effects of protein consumption. Nine out of 11 trials were in favour of consuming protein for reducing isometric strength loss compared to control products at ≥1 time-point. Likewise, only one trial failed to demonstrate a positive effect of protein for attenuating post-exercise [CK] elevations. Protein consumption is unlikely beneficial for reducing post-exercise muscle soreness in young males, as zero-small effects were observed (ES range = 0.004–0.195). This review could not establish the impact of protein supplementation on EIMD in females due to a lack of studies conducted with females or both sexes.

Despite its frequent assessment, the efficacy of protein consumption for muscle soreness management is confounding. Less than half of trials reviewed reported a benefit of protein for reducing post-exercise muscle soreness at 48 h. These conflicting data reflect the existing limited understanding of the mechanisms of exercise-induced muscle soreness, alongside its subjectivity and susceptibility to other physiological and psychological influencers (e.g., mood, sleep quality, hormonal status) [65]. However, this review identified that males untrained in resistance exercise are more likely to respond positively to protein supplementation than trained males. Similarly, protein supplementation more frequently reduced symptoms of muscle soreness following concentric than eccentric exercise. Therefore, both training status and muscle contraction type may influence muscle soreness responses to protein supplementation. Investigating these factors may allude to muscle soreness mechanisms, although current understanding is hindered by the varied assessment methods used, for example, different rating scales, participant positioning, pressure algometry, muscle palpation, passively, or actively with mixed forms of activity. Such inconsistencies might explain why muscle soreness is argued to poorly reflect EIMD [66]. Until optimal and consistent methods for assessing muscle soreness are employed, data should be treated cautiously.

Post-exercise muscle strength decrements debilitate future exercise quality for up to 7 days [13, 14]. This review has demonstrated that peri-exercise protein consumption can reduce muscle strength loss and accelerate the recovery of muscle function. Therefore, high-quality exercise may be resumed faster with the aid of protein consumption, compared to carbohydrate-based or no peri-exercise nutritional strategy. Currently, the use of protein as an exercise nutrition strategy is recommended for muscle recovery, repair, and growth, due to its stimulatory effects on post-exercise MPS rates [67], which are augmented by protein consumption relative to exercise alone [35]. It follows that peri-exercise protein consumption may be recommended as a multi-purpose nutritional aid – assisting in the management of muscle damage and repair processes. This dual-target strategy could help lift financial, time, and resource constraints, as opposed to following multiple dietary and/or physiotherapeutic strategies serving individual purposes.

It is unclear how protein supplementation may reduce muscle strength decrements following exercise and here, protein ingestion was only beneficial for isometric MVC at 96 h post-exercise. One explanation is that MPS is augmented by protein relative to carbohydrate ingestion during the later (72–168 h) but not earlier (24–72 h) recovery period after EIMD [68]. By this means, repair and remodelling of muscle proteins and restoration of muscle function may occur at an accelerated rate with protein supplementation. However, it is difficult to explain why protein supplementation benefited isokinetic but not isometric MVC at 24–72 h post-exercise. These outcomes appear predominantly due to one influential study [63], though nonetheless could relate to the magnitude of strength decline, which was typically lower for isokinetic compared with isometric MVC. The pathways by which protein ingestion acts to attenuate EIMD warrant investigation, though notably, factors other than post-exercise amino acid availability play a role [68].

Much research on protein nutrition, particularly related to MPS, has sought to establish the optimal type, dose, and timing of protein consumption to maximally stimulate post-exercise MPS rates [69]. Although, this has not been the case for EIMD. The present review identified no discernible effects of protein ingestion timing, type, dosage (ranged 5–104 g), or days of supplementation on EIMD (supplementary fig. 1). However, other inter-study differences in methodological design, for example the exercise protocol, sample demographics, and measurement tools may limit the ability to compare protein supplementation protocols between trials.

This review identified few trials that compared protein supplementation strategies while being matched for other methodological detail. White et al. [43] provided untrained males with 23 g whey protein plus 75 g carbohydrate before or after eccentric leg extension exercise, which had no impact on EIMD, irrespective of supplementation timing. Likewise, there were no differences in EIMD between groups of untrained males consuming a large dose (1.5 g•kgBM^−1^) of whey protein pre, post, or pre and post exercise [42]. In a series of experiments with comparable methodological design, Cockburn and colleagues examined the impact of various milk protein feeding strategies on EIMD in trained males performing leg-based resistance exercise. These authors found no significant interactions between EIMD markers and milk protein timing (pre, post, or 24 h post-exercise), dosage (17 or 34 g), or type (milk or flavoured milk plus carbohydrate) [36, 38, 39]. Similarly, ingesting flavoured milk relative to an isonitrogenous dose of whey protein hydrolysate did not impact muscle damage following whole-body resistance exercise in trained males [70]. Conversely, Buckley et al. [44] indicate that the type of ingested protein influences EIMD.

Buckley and colleagues [44] noted that maximal isometric strength was preserved in untrained males at 6–24 h following maximal eccentric exercise when hydrolysed whey protein, but not isolated whey protein or flavoured water, was consumed post-exercise. This finding is unusual, especially as there were no between-group differences in peak strength decrements at 0–2 h post-exercise, nor in other EIMD markers. Another anomaly is the tendency for maximal strength to undergo a second decrease from 6 to 24 h following whey isolate ingestion, meanwhile maximal strength of the placebo group began returning to baseline. The authors propose that the hydrolysed whey protein accelerated strength recovery relative to non-hydrolysed protein by means of stimulating muscle repair processes. However, this theory seems unlikely, given that isolated whey protein stimulates increased post-exercise MPS rates [71–74]. Furthermore, a similar study observed comparable isometric strength reductions and recovery rates after eccentric exercise with ingested whey hydrolysate, whey isolate, and flavoured water [45]. Supporting data [60] make it challenging to explain the outcomes of Buckley [44]; thus, the impact of protein hydrolysis on EIMD warrants further investigation. To ascertain the importance of protein feeding type, timing, and dosage for the management of EIMD, further studies with comparable methodologies are required. Due to the apparent lack of difference between isolated and whole-food sources of protein, future studies should adopt a food-first approach where feasible.

The food-first approach aids the achievement of multiple nutrient requirements, however meeting protein intake goals using this approach may be challenging for some protein types. Accordingly, the ‘food first but not always food only’ approach is advocated [75]. Plant-based proteins present a challenge, as they necessitate consumption of larger food volumes to achieve protein requirements. For example, 20 g of milk protein can be obtained through ~200 g of dairy yoghurt, while ~500 g of soya yoghurt is required to obtain 20 g of soy protein. Alternatively, a single-serve of isolated soy protein conveniently provides an isonitrogenous dose. Plant-based diets are growing in popularity, due to various health, environmental, ethical, and economic benefits [76]. Although, the impact of plant-based proteins on EIMD is uncertain. Three studies considered the impact of plant- versus animal- based proteins on resistance EIMD in untrained males in the present review [41, 58, 60]. Hasegawa and colleagues [58] provided participants with 20 g of egg white or isolated soy protein or water preceding whole-body resistance exercise. Serum [CK] and muscle soreness significantly increased 30 min and 24 h post-exercise, respectively, with no between-group differences. Nieman et al. [41] found that consuming ~24 g of isolated whey, but not pea, protein before and after whole-body exercise, and pre-sleep for 4 d, attenuated serum [CK] elevations at 72–96 h post-exercise compared to water consumption. Nonetheless, relative to water, neither protein source reduced muscle strength, endurance, and power decrements or muscle soreness. Here, the ineffectiveness of plant-based proteins for reducing EIMD may be attributed to their single-source origin. Plant-based proteins, including soy, rice, and wheat, have been scrutinised as inferior in quality to animal-based proteins, due to their lower essential amino acid content [77] and bioavailability [78]. Ingesting a larger dose [79] or a blend [80, 81] of plant-based proteins provides the amino acid profile required to stimulate increased MPS rates. In this review, only one study [60] compared the effect of a plant-based protein blend on EIMD with whey protein isolate, whey protein hydrolysate, and a non-isoenergetic control. EIMD was unaffected by peri-exercise ingestion of a 25–40 g protein dose, irrespective of source, which is perhaps partly due to equivalent daily total protein intakes between groups. Further investigation of plant- versus animal- based proteins and single-source versus blended plant-based proteins from isolated and whole-food sources is needed to determine the relevance of protein quality in EIMD.

The present findings on the efficacy of ingested protein for muscle function restoration following resistance exercise are consistent with Davies and colleagues [27]. This meta-analysis (*n* = 13 trials) reported small-medium beneficial effects of whey protein consumption <24–96 h post-exercise. However, peak isometric knee extensor strength was the only outcome considered, and without corroboration from other EIMD markers these data have narrow application. Further, varied control groups were included (water, carbohydrate, milk, and collagen proteins), making inter-trial generalisability unreasonable. Conversely, the systematic review by Pasiakos et al. [31] examined the impact of a range of protein- and amino acid- based supplements on EIMD outcomes (muscle function, soreness, CK, LDH, Mb) for up to several weeks following endurance or resistance exercise. These authors found minimal evidence supporting a benefit of protein supplementation for post-exercise recovery of muscle function and soreness. However, they acknowledge that divergencies in study design regarding protein supplementation and exercise protocols limit their observations. In contrast, the present review identified an overall advantage to consuming protein on muscle function, which may reflect the tighter study inclusion criteria (resistance exercise only, separation of muscle functional markers, exclusion of amino acid-based supplements). Seemingly, broad criteria for study inclusion may mask beneficial effects of protein supplementation for EIMD, especially when small sample sizes prevent sub-group analysis.

## Limitations

Several limitations may have affected the outcomes and application of the present review. From the pool of studies, 5 were considered low/fair quality and therefore susceptible to bias, which can exaggerated treatment effects [82]. The main PEDro criteria that studies failed to meet were lack of double blinding, which can induce bias; completion of ≥1 outcome by 85% of participants; and receipt of allocated treatment. However, failure to meet these criteria was often assumed, due to a lack of methods reporting. Other limitations arose from the supplementation strategies and study designs employed. Control supplements were varied (artificial sweetener, water, flavoured water, electrolytes, glucose, maltodextrin, dextrose, and no supplement [42, 62, 83, 84]), and mostly, not isoenergetic to protein supplements; thus, energy/carbohydrate content may have confounded protein effects. A range of protein doses were given, potentially increasing heterogeneity of the study pool. Two studies [42, 85] prescribed protein dose relative to body mass (1.5 g•kgBM^−1^) resulting in large doses (~104 g), although most studies provided a standard dose (17–42 g).

Eight studies were possibly limited by adopting crossover designs. Due to RBEs associated with EIMD [16–18], responses to repeated exercise were likely attenuated, particularly in untrained participants [57, 58, 84] and with insufficient washout periods (1–2 wk) [57, 58, 61, 62, 86, 87]. Notwithstanding, all crossover studies counterbalanced treatment order, which should limit order effects and the impact of RBEs.

Regarding the meta-analyses, ESs were not obtained for all variables in each trial due to insufficient data reporting. However, no apparent differences existed in the outcomes of included or excluded trials. Data extraction from figures may have been inaccurate, resulting in over/underestimated treatment effects. Furthermore, when sample size was not reported for each variable and time-point, a consistent sample size was assumed, which if inaccurate could alter true effects. Variables with different assessment methods (e.g., active and passive soreness) were pooled to maximise *k* for meta-analyses; however, this might impact overall treatment effects. This review considered only four variables, thus providing scope for future meta-analyses to examine protein supplementation effects on other markers of EIMD. Moreso, due to its large-scale, this review did not consider amino acid-based supplements, which may offer beneficial sub-analysis. Finally, as the study samples were 94% young male, the outcomes of this review may be inapplicable to older adults and females.

### Future directions

The limited understanding of the impact of protein supplementation for resistance EIMD management in females should be addressed by conducting high-quality research with females or both sexes. Additional investigation of various protein types (particularly plant-based), timing, and dosing strategies would help inform protein nutrition guidelines for EIMD management. Establishing optimal methods for assessing EIMD in experimental models requires investigation, as methodological inconsistencies across current studies are hindering knowledge progression of EIMD mechanisms and management strategies. To benefit future research, standardised methodologies (e.g., consistent measures, measurement time-points, assessment tools) should be practiced, increasing the generalisability and application of outcomes, and data inclusion in topical reviews and meta-analyses. Where feasible, cross-over designs with sufficient wash-out period and, when relevant, unilateral limb models should be employed to limit heterogeneity. Furthermore, data reporting and transparency issues are limiting study inclusion in meta-analyses and obstructing accurate and representative conclusions being drawn. Accordingly, a framework is proposed outlining data reporting guidance to increase inclusion of primary data in meta-analyses (Table 3).

**Table 3:** A framework of data reporting guidelines for primary research to increase inclusion in meta-analyses.

**Proposed guidelines for data reporting in primary research**
Authors should provide supplementary data files of all gathered data where feasible
When measures are conducted at multiple time-points, authors should strive to report the mean change and variance between time-points (not just mean and variance data at each time-point)
Where feasible, baseline data for all measures should be obtained and reported
The acquired sample size for each variable at each time-point should be reported (these data could be included in figure legends or supplementary files)
Studies conducted with participants of both sexes should report male and female data separately (even if the analysis of sex differences is not an outcome of the study)
Authors should report the methods used relating to study quality, e.g., randomisation, blinding, allocation concealment. If no consideration has been given to these methodological factors, this should be stated.

## Conclusions

This systematic review with meta-analysis demonstrated that, in young males, peri-exercise protein consumption reduces maximal strength decrements and lowers [CK] following acute resistance exercise but does not benefit muscle soreness. These outcomes are seemingly unaffected by the type, timing, frequency, and dose of ingested protein, though may be affected by the exercise protocol and sample training status, with further examination required. This review identified an absence of female-focussed research and a limited number of studies examining plant-based protein sources, which warrants future research priority. Developing evidence-based EIMD management strategies is impeded by methodological inconsistencies across studies, particularly pertaining to EIMD assessment methods. This review highlights the need for standardised and transparent data reporting in EIMD research and proposes a guiding framework.

## Supplementary information

Table S1

Figure S1

PRISMA abstract checklist

PRISMA checklist

## Data Availability

All data synthesised are presented within the manuscript or are available from the corresponding author upon request.

## References

[1] Bull FC, Al-Ansari SS, Biddle S, Borodulin K, Buman MP, Cardon G (2020). World Health Organization 2020 guidelines on physical activity and sedentary behaviour. Br J Sports Med.

[2] Tan B (1999). Manipulating resistance training program variables to optimize maximum strength in men: a review. J Strength Cond Res.

[3] Schoenfeld (2010). The mechanisms of muscle hypertrophy and their application to resistance training. J Strength Cond Res.

[4] Anderson, Behm DG (2005). The impact of instability resistance training on balance and stability. Sports Med.

[5] Eriksson J, Tuominen J, Valle T, Sundberg S, Sovijärvi A, Lindholm H (1998). Aerobic endurance exercise or circuit-type resistance training for individuals with impaired glucose tolerance?. Horm Metab Res.

[6] Layne JE, Nelson ME (1999). The effects of progressive resistance training on bone density: a review. Med Sci Sports Exerc.

[7] Staublr WT (1989). Eccentric action of muscles: physiology, injury, and adaptation. Exerc sport Sci Rev.

[8] Proske U, Morgan DL (2001). Muscle damage from eccentric exercise: mechanism, mechanical signs, adaptation and clinical applications. J Physiol.

[9] Warren GL, Lowe DA, Hayes DA, Karwoski CJ, Prior BM, Armstrong RB (1993). Excitation failure in eccentric contraction-induced injury of mouse soleus muscle. J Physiol.

[10] Nosaka K, Newton M (2002). Difference in the magnitude of muscle damage between maximal and submaximal eccentric loading. J Strength Cond Res.

[11] Nosaka M, Newton, Sacco P (2002). Muscle damage and soreness after endurance exercise of the elbow flexors. Med Sci Sports Exerc.

[12] Hesselink MK, Kuipers H, Geurten P, Van H (1996). Straaten, Structural muscle damage and muscle strength after incremental number of isometric and forced lengthening contractions. J Muscle Res Cell Motil.

[13] Farup J, Rahbek SK, Knudsen IS, de Paoli F, Mackey AL, Vissing K (2014). Whey protein supplementation accelerates satellite cell proliferation during recovery from eccentric exercise. Amino Acids.

[14] Byrne C, Eston R (2002). Maximal-intensity isometric and dynamic exercise performance after eccentric muscle actions. J Sports Sci.

[15] Damas SM, Phillips CA, Libardi FC, Vechin ME, Lixandrao PR, Jannig (2016). Resistance training-induced changes in integrated myofibrillar protein synthesis are related to hypertrophy only after attenuation of muscle damage. J Physiol-Lond.

[16] Fridén J, Seger J, Sjöström M, Ekblom B (1983). Adaptive response in human skeletal muscle subjected to prolonged eccentric training. Int J Sports Med.

[17] Schwane JA, Armstrong RB (1983). Effect of training on skeletal muscle injury from downhill running in rats. J Appl Physiol Respir Environ Exerc Physiol.

[18] Hough T (1902). Ergographic studies in muscular soreness. Am Phys Educ Rev.

[19] Malm C, Nyberg P, Engstrom M, Sjodin B, Lenkei R, Ekblom B (2000). Immunological changes in human skeletal muscle and blood after eccentric exercise and multiple biopsies. J Physiol.

[20] Clarkson PM, Hubal MJ (2002). Exercise-induced muscle damage in humans. Am J Phys Med Rehabil.

[21] Warren DA, Lowe, Armstrong RB (1999). Measurement tools used in the study of eccentric contraction-induced injury. Sports Med.

[22] Morton JP, Atkinson G, MacLaren DPM, Cable NT, Gilbert G, Broome C (2005). Reliability of maximal muscle force and voluntary activation as markers of exercise-induced muscle damage. Eur J Appl Physiol.

[23] Nosaka K, Clarkson PM (1996). Variability in serum creatine kinase response after eccentric exercise of the elbow flexors. Int J sports Med.

[24] Damas F, Nosaka K, Libardi CA, Chen TC, Ugrinowitsch C (2016). Susceptibility to exercise-induced muscle damage: a cluster analysis with a large sample. Int J Sports Med.

[25] Torres R, Ribeiro F, Alberto Duarte J, Cabri JMH (2012). Evidence of the physiotherapeutic interventions used currently after exercise-induced muscle damage: Systematic review and meta-analysis. Phys Ther Sport.

[26] Dupuy O, Douzi W, Theurot T, Bosquet L, Dugué B. An evidence-based approach for choosing post-exercise recovery techniques to reduce markers of muscle damage, soreness, fatigue, and inflammation: a systematic review with meta-analysis. Front Physiol. 2018;9:403. 10.3389/fphys.2018.00403 PMC5932411 29755363

[27] Davies RW, Carson BP, Jakeman PM (2018). The effect of whey protein supplementation on the temporal recovery of muscle function following resistance training: a systematic review and meta-analysis. Nutrients.

[28] Harty PS, Cottet ML, Malloy JK, Kerksick CM (2019). Nutritional and supplementation strategies to prevent and attenuate exercise-induced muscle damage: a brief review. Sports Med - Open.

[29] Rahimi MH, Shab-Bidar S, Mollahosseini M, Djafarian K (2017). Branched-chain amino acid supplementation and exercise-induced muscle damage in exercise recovery: A meta-analysis of randomized clinical trials. Nutrition.

[30] Rahimi MH, Mohammadi H, Eshaghi H, Askari G, Miraghajani M (2018). The effects of Beta-Hydroxy-Beta-Methylbutyrate supplementation on recovery following exercise-induced muscle damage: a systematic review and meta-analysis. J Am Coll Nutr.

[31] Pasiakos SM, Lieberman HR, McLellan TM (2014). Effects of protein supplements on muscle damage, soreness and recovery of muscle function and physical performance: a systematic review. Sports Med.

[32] Fouré A, Bendahan D (2017). Is branched-chain amino acids supplementation an efficient nutritional strategy to alleviate skeletal muscle damage? A systematic review. Nutrients.

[33] Poulios A, Georgakouli K, Draganidis D, Deli CK, Tsimeas PD, Chatzinikolaou A (2019). Protein-based supplementation to enhance recovery in team sports: what is the evidence?. J Sports Sci Med.

[34] Tipton K (2008). Protein for adaptations to exercise training. Eur J Sport Sci.

[35] Moore MJ, Robinson JL, Fry JE, Tang EI, Glover SB, Wilkinson (2009). Ingested protein dose response of muscle and albumin protein synthesis after resistance exercise in young men. Am J Clin Nutr.

[36] Cockburn E, Hayes PR, French DN, Stevenson E, St Clair Gibson A (2008). Acute milk-based protein–CHO supplementation attenuates exercise-induced muscle damage. Appl Physiol Nutr Metab.

[37] Draganidis D, Chondrogianni N, Chatzinikolaou A, Terzis G, Karagounis LG, Sovatzidis A (2017). Protein ingestion preserves proteasome activity during intense aseptic inflammation and facilitates skeletal muscle recovery in humans. Br J Nutr.

[38] Cockburn, Stevenson E, Hayes PR, Robson-Ansley P, Howatson G (2010). Effect of milk-based carbohydrate-protein supplement timing on the attenuation of exercise-induced muscle damage. Appl Physiol, Nutr Metab.

[39] Cockburn E, Robson-Ansley P, Hayes PR, Stevenson E (2012). Effect of volume of milk consumed on the attenuation of exercise-induced muscle damage. Eur J Appl Physiol.

[40] Rankin P, Stevenson E, Cockburn E (2015). The effect of milk on the attenuation of exercise-induced muscle damage in males and females. Eur J Appl Physiol.

[41] Nieman D, Zwetsloot KA, Simonson AJ, Hoyle AT, Wang X, Nelson HK (2020). Effects of whey and pea protein supplementation on post-eccentric exercise muscle damage: a randomized trial. Nutrients.

[42] Kim J, Lee C, Lee J (2017). Effect of timing of whey protein supplement on muscle damage markers after eccentric exercise. J Exerc Rehabil.

[43] White JP, Wilson JM, Austin KG, Greer BK, St. John N, Panton LB. Effect of carbohydrate-protein supplement timing on acute exercise-induced muscle damage. J Int Soc Sports Nutr, 2008;5:1. 10.1186/1550-2783-5-5 PMC2288590 18284676

[44] Buckley JD, Thomson RL, Coates AM, Howe PRC, DeNichilo MO, Rowney MK (2010). Supplementation with a whey protein hydrolysate enhances recovery of muscle force-generating capacity following eccentric exercise. J Sci Med Sport.

[45] Dale M, Thomson R, Coates A, Howe PR, Brown A, Buckley JD (2015). Protein hydrolysates and recovery of muscle damage following eccentric exercise. Funct Foods Health Dis.

[46] Moher D, Liberati A, Tetzlaff J, Altman DG, Group P (2009). Preferred reporting items for systematic reviews and meta-analyses: the PRISMA statement. PLoS Med.

[47] Ouzzani M, Hammady H, Fedorowicz Z, Elmagarmid A (2016). Rayyan—a web and mobile app for systematic reviews. Syst Rev.

[48] Schoenfeld J, Grgic D, Ogborn, Krieger JW (2017). Strength and hypertrophy adaptations between low- vs. high-load resistance training: a systematic review and meta-analysis. J Strength Cond Res.

[49] Maher CG, Sherrington C, Herbert RD, Moseley AM, Elkins M (2003). Reliability of the PEDro scale for rating quality of randomized controlled trials. Phys Ther.

[50] Elkins MR, Herbert RD, Moseley AM, Sherrington C, Maher C (2010). INVITED COMMENTARY: Rating the quality of trials in systematic reviews of physical therapy interventions. Cardiopulm Phys Ther J.

[51] Borenstein, M, LV Hedges, JP Higgins, and HR Rothstein, *Introduction to meta-analysis*. 2021: John Wiley & Sons.

[52] Higgins JP, Thompson SG, Deeks JJ, Altman DG (2003). Measuring inconsistency in meta-analyses. BMJ.

[53] Cohen J (1992). A power primer. Psychol Bull.

[54] Paulsen UR, Mikkelsen T, Raastad, Peake JM (2012). Leucocytes, cytokines and satellite cells: what role do they play in muscle damage and regeneration following eccentric exercise?. Exerc Immunol Rev.

[55] Baty JJ, Hwang H, Ding Z, Bernard JR, Wang B, Kwon B (2007). The effect of a carbohydrate and protein supplement on resistance exercise performance, hormonal response, and muscle damage. J Strength Cond Res.

[56] Samadi A, Gaeini AA, Kordi MR, Rahimi M, Rahnama N, Bambaeichi E (2012). Effect of various ratios of carbohydrate-protein supplementation on resistance exercise-induced muscle damage. J Sports Med Phys Fit.

[57] Burnley ECD, Olson AN, Sharp RL, Baier SM, Alekel DL (2010). Impact of protein supplements on muscle recovery after exercise-induced muscle soreness. J Exerc Sci Fit.

[58] Hasegawa Y, Mekata Y, Ayaka S, Yuri Y, Takahiro Y, Maya H (2014). Effect of egg white protein supplementation prior to acute resistance training on muscle damage indices in untrained Japanese men. Montenegrin J Sports Sci Med.

[59] Hoffman JR, Ratamess NA, Tranchina CP, Rashti SL, Kang J, Faigenbaum AD (2010). Effect of a proprietary protein supplement on recovery indices following resistance exercise in strength/power athletes. Amino Acids.

[60] Saracino PG, Saylor HE, Hanna BR, Hickner RC, Kim JS, Ormsbee MJ (2020). Effects of pre-sleep whey vs. Plant-based protein consumption on muscle recovery following damaging morning exercise. Nutrients.

[61] Grubic TJ, Sowinski RJ, Nevares BE, Jenkins VM, Williamson SL, Reyes AG, et al. Comparison of ingesting a food bar containing whey protein and isomalto-oligosaccharides to carbohydrate on performance and recovery from an acute bout of resistance-exercise and sprint conditioning: an open label, randomized, counterbalanced, crossover pilot study. J Int Soc Sports Nutr. 2019;16:1. 10.1186/s12970-019-0301-z PMC6693099 31409363

[62] West DWD, Sawan SA, Mazzulla M, Williamson E, Moore DR. Whey protein supplementation enhances whole body protein metabolism and performance recovery after resistance exercise: a double-blind crossover study. Nutrients, 2017;9:725. 10.3390/nu9070735 PMC5537849 28696380

[63] Philpott JD, Donnelly C, Walshe IH, MacKinley EE, Dick J, Galloway SDR (2018). Adding fish oil to whey protein, leucine, and carbohydrate over a six-week supplementation period attenuates muscle soreness following eccentric exercise in competitive soccer players. Int J Sport Nutr Exerc Metab.

[64] Cockburn PG, Bell, Stevenson E (2013). Effect of milk on team sport performance after exercise-induced muscle damage. Med Sci Sports Exerc.

[65] Melzack R (1982). Recent concepts of pain. J Med.

[66] Nosaka M, Newton, Sacco P (2002). Delayed-onset muscle soreness does not reflect the magnitude of eccentric exercise-induced muscle damage. Scand J Med Sci Sports.

[67] Tipton, Ferrando AA, Phillips SM, Doyle Jr D, Wolfe RR (1999). Postexercise net protein synthesis in human muscle from orally administered amino acids. Am J Physiol-Endocrinol Metab.

[68] Pavis GF, Jameson TSO, Dirks ML, Lee BP, Abdelrahman DR, Murton AJ (2021). Improved recovery from skeletal muscle damage is largely unexplained by myofibrillar protein synthesis or inflammatory and regenerative gene expression pathways. Am J Physiol-Endocrinol Metab.

[69] Witard OC, Wardle SL, Macnaughton LS, Hodgson AB, Tipton KD (2016). Protein considerations for optimising skeletal muscle mass in healthy young and older adults. Nutrients.

[70] Gee TI, Woolrich TJ, Smith MF (2019). Effectiveness of whey protein hydrolysate and milk-based formulated drinks on recovery of strength and power following acute resistance exercise. J Hum Kinet.

[71] Burd NA, Yang Y, Moore DR, Tang JE, Tarnopolsky MA, Phillips SM (2012). Greater stimulation of myofibrillar protein synthesis with ingestion of whey protein isolate v. micellar casein at rest and after resistance exercise in elderly men. Br J Nutr.

[72] Yang Y, Breen L, Burd NA, Hector AJ, Churchward-Venne TA, Josse AR (2012). Resistance exercise enhances myofibrillar protein synthesis with graded intakes of whey protein in older men. Br J Nutr.

[73] Macnaughton LS, Wardle SL, Witard OL, McGlory C, Hamilton DL, Jeromson S, et al. The response of muscle protein synthesis following whole-body resistance exercise is greater following 40 g than 20 g of ingested whey protein. Physiol Rep. 2016;4:e12893. 10.14814/phy2.12893 PMC4985555 27511985

[74] Borack MS, Reidy PT, Husaini SH, Markofski MM, Deer RR, Richison AB (2016). Soy-dairy protein blend or whey protein isolate ingestion induces similar postexercise muscle mechanistic target of rapamycin complex 1 signaling and protein synthesis responses in older Men. The. J Nutr.

[75] Close GL, A Kasper, N Walsh, and R Maughan, “Food First but Not Always Food Only”: Recommendations for using dietary supplements in sport. Int J Sport Nutr Exercise Metab., 2022. 10.1123/ijsnem.2021-0335 35279015

[76] Fehér A, Gazdecki M, Véha M, Szakály M, Szakály Z (2020). A comprehensive review of the benefits of and the barriers to the switch to a plant-based diet. Sustainability.

[77] Gorissen S, Crombag JJR, Senden JMG, Waterval WAH, Bierau J, Verdijk LB (2018). Protein content and amino acid composition of commercially available plant-based protein isolates. Amino Acids.

[78] van Vliet S, Burd NA, van Loon LJ (2015). The skeletal muscle anabolic response to plant- versus animal-based protein consumptione. J Nutr.

[79] Gorissen S, Horstman AM, Franssen R, Crombag JJ, Langer H, Bierau J (2016). Ingestion of wheat protein increases in vivo muscle protein synthesis rates in healthy older men in a randomized trial. J Nutr.

[80] Kouw IWK, Pinckaers PJM, Le Bourgot C, van Kranenburg JMX, Zorenc AH, de Groot LCPGM, et al. Ingestion of an ample amount of meat substitute based on a lysine-enriched, plant-based protein blend stimulates postprandial muscle protein synthesis to a similar extent as an isonitrogenous amount of chicken in healthy, young men. Br J Nutr. 2021;128:1955–65. 10.1017/S0007114521004906 34881688

[81] Pinckaers PJM, Kouw IWK, Gorissen SHM, Houben LHP, Senden JM, Wodzig WKHW, et al. The muscle protein synthetic response to the ingestion of a plant-derived protein blend does not differ from an equivalent amount of milk protein in healthy, young males. J Nutr. 2022;nxac222. 10.1093/jn/nxac222 PMC9839989 36170964

[82] Pildal J, Hróbjartsson A, Jørgensen K, Hilden J, Altman D, Gøtzsche P (2007). Impact of allocation concealment on conclusions drawn from meta-analyses of randomized trials. Int J Epidemiol.

[83] Karakus M, Akkurt S (2020). The effect of use of protein supplements on muscle damage. Prog Nutr.

[84] Hirose N, Sato M, Yanagisawa O, Fukubayashi T (2013). Milk peptide intake may decrease muscle damage after eccentric exercise. Int J Sport Health Sci.

[85] Cooke MB, Rybalka E, Stathis CG, Cribb PJ, Hayes A (2010). Whey protein isolate attenuates strength decline after eccentrically-induced muscle damage in healthy individuals. J Int Soc Sports Nutr.

[86] Bird SP, Mabon T, Pryde M, Feebrey S, Cannon J (2013). Triphasic multinutrient supplementation during acute resistance exercise improves session volume load and reduces muscle damage in strength-trained athletes. Nutr Res.

[87] Naclerio F, Larumbe-Zabala E, Cooper K, Seijo M (2020). Effects of a multi-ingredient beverage on recovery of contractile properties, performance, and muscle soreness after hard resistance training sessions. J Strength Cond Res.

