# Supplementary material for: The impact of dietary protein supplementation on recovery from resistance exercise-induced muscle damage: A systematic review with meta-analysis
Source: Eur J Clin Nutr. 2022 Dec 13;77(8):767–83. doi: 10.1038/s41430-022-01250-y (PMC10393778; doi:10.1038/s41430-022-01250-y)
Supplement: Supplementary file 1 — Table S1 [file 41430_2022_1250_MOESM1_ESM.docx]

**Table S1** Magnitude of exercise-induced muscle damage

| Reference | Supplement group | Peak change from baseline (%) | EIMD magnitude | Hedges’ *g* effect size |
| --- | --- | --- | --- | --- |
| **Isometric MVC** |  |  |  |  |
| Buckley et al. [44] | Protein _(a)_ | -19.3 ± 8.6 | Mild | 0.05 [-0.06, 0.15] |
| Buckley et al. [44] | Protein _(b)_ | -30.2 ± 8.8 | Moderate | -0.53 [-0.84, -0.23] |
| Buckley et al. [44] | Control | -20.2 ± 23.7 | Moderate |  |
| Cooke et al. [65] | Protein | -12.5 ± 13.8 | Mild | 0.64 [0.26, 1.02] |
| Cooke et al. [65] | Control | -21.0 ± 11.0 | Moderate |  |
| Dale et al. [45] | Protein _(a)_ | -22.4 ± 4.9 | Moderate | -0.42 [-0.65, -0.19] |
| Dale et al. [45] | Protein _(b)_ | -23.5 ± 3.4 | Moderate | -0.82 [-1.24, -0.40] |
| Dale et al. [45] | Control | -20.4 ± 3.8 | Moderate |  |
| Draganidis et al. [37] | Protein | -27.4^*^ | Moderate | 0.91 [0.51, 1.31] |
| Draganidis et al. [37] | Control | -39.6^*^ | Moderate |  |
| Farup et al. [13] | Protein | -24.2 ± 18.7 | Moderate | 0.27 [0.06, 0.48] |
| Farup et al. [13] | Control | -28.7 ± 12.8 | Moderate |  |
| Ives et al. [68] | Protein | -22.1 ± 10.4 | Moderate | 0.25 [0.07, 0.43] |
| Ives et al. [68] | Control | -25.8 ± 17.6 | Moderate |  |
| Kim et al. [42] | Protein _(a)_ | -56.7 ± 15.1 | Severe | 0.31 [0.04, 0.58] |
| Kim et al. [42] | Protein _(b)_ | -59.7 ± 20.0 | Severe | 0.11 [-0.05, 0.28] |
| Kim et al. [42] | Protein _(c)_ | -60.8 ± 18.8 | Severe | 0.06 [-0.06, 0.18] |
| Kim et al. [42] | Control | -62.2 ± 12.4 | Severe |  |
| **Isokinetic MVC** |  |  |  |  |
| Cockburn et al. [38] | Protein _(a)_ | -22.0 ± 14.0 | Moderate | 0.06 [-0.06, 0.19] |
| Cockburn et al. [38] | Protein _(b)_ | -7.0 ± 30.0 | Mild | 0.46 [0.13, 0.80] |
| Cockburn et al. [38] | Protein _(c)_ | -16.0 ± 24.0 | Mild | 0.3 [0.03, 0.57] |
| Cockburn et al. [38] | Control | -27.0 ± 42.0 | Moderate |  |
| Cockburn et al. [39] | Protein _(a)_ | -14.0 ± 15.5 | Mild | 0.13 [ -0.05, 0.31] |
| Cockburn et al. [39] | Protein _(b)_ | -12.5 ± 14.0 | Mild | 0.2 [-0.02, 0.42] |
| Cockburn et al. [39] | Control | -17.0 ± 26.5 | Mild |  |
| Cooke et al. [65] | Protein | -8.6 ± 17.7 | Mild | 0.37 [0.08, 0.66] |
| Cooke et al. [65] | Control | -14.0 ± 7.1 | Mild |  |
| Philpott et al. [71] | Protein | -9.7 ± 9.6 | Mild | 1.34 [0.84, 1.85] |
| Philpott et al. [71] | Control | -30.0 ± 5.8 | Moderate |  |
| Rankin et al. [40] | Protein | -11.4 ± 25.4 | Mild | 0.7 [0.41, 0.98] |
| Rankin et al. [40] | Control | -29.1 ± 24.2 | Moderate |  |

*The magnitude of exercise-induced muscle damage (EIMD) is based on the peak percent reduction from baseline in isometric (k = 11) and isokinetic (k = 8) maximal voluntary contraction (MVC) and categorised as mild (<20% MVC reduction), moderate (20-50% MVC reduction), or severe (>50% MVC reduction) as per Paulsen et al. [54]. Only trials included in the meta-analyses are presented alongside the Hedges’ g effect size at the time-point of peak MVC change; data expressed as mean ± standard deviation (SD); *SD not reported in manuscript (g calculated from reported ES).*
