# Supplementary material for: The impact of dietary protein supplementation on recovery from resistance exercise-induced muscle damage: A systematic review with meta-analysis
Source: Eur J Clin Nutr. 2022 Dec 13;77(8):767–83. doi: 10.1038/s41430-022-01250-y (PMC10393778; doi:10.1038/s41430-022-01250-y)
Supplement: Supplementary file 2 — Figure S1 [file 41430_2022_1250_MOESM2_ESM.docx]

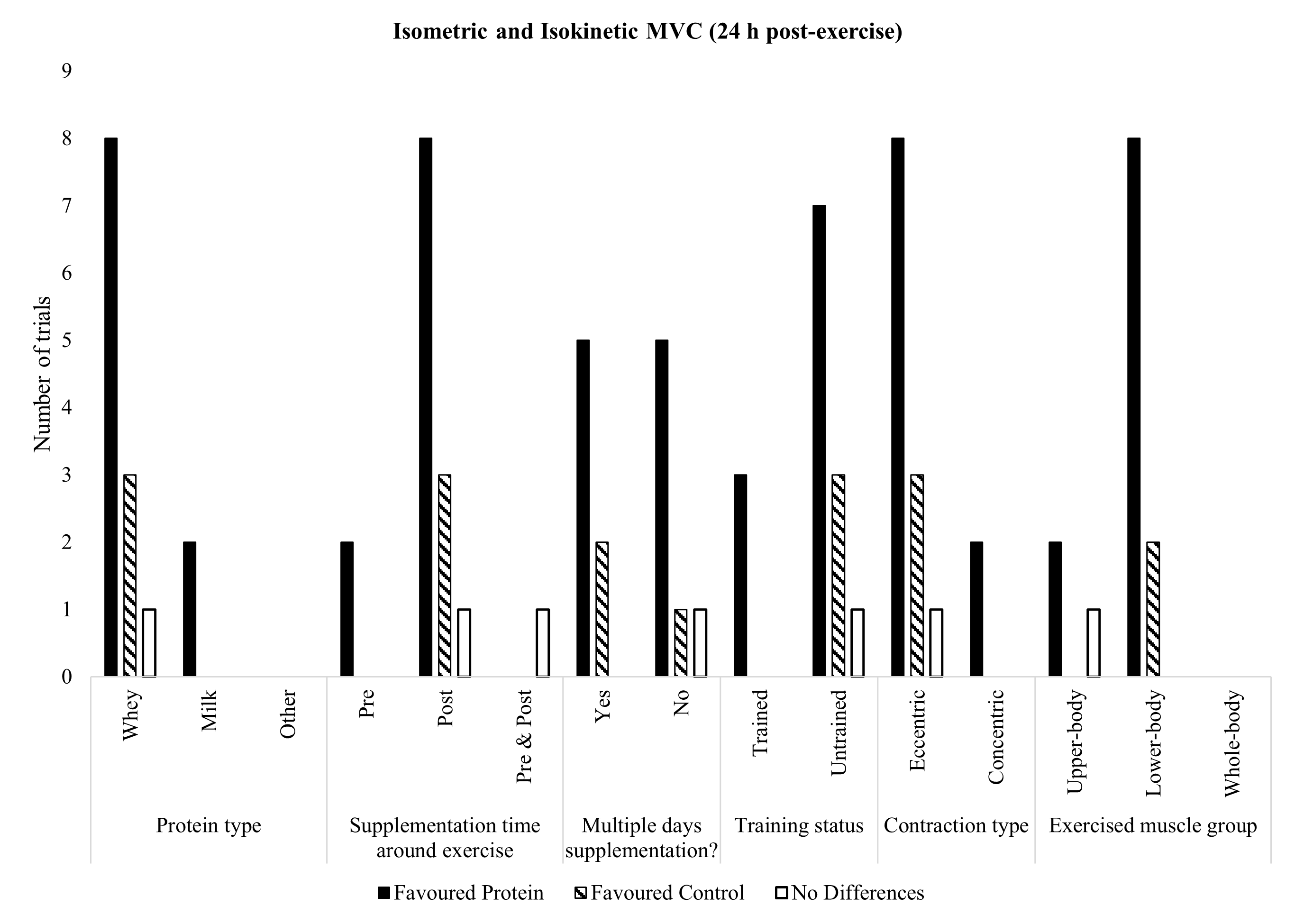


***a***


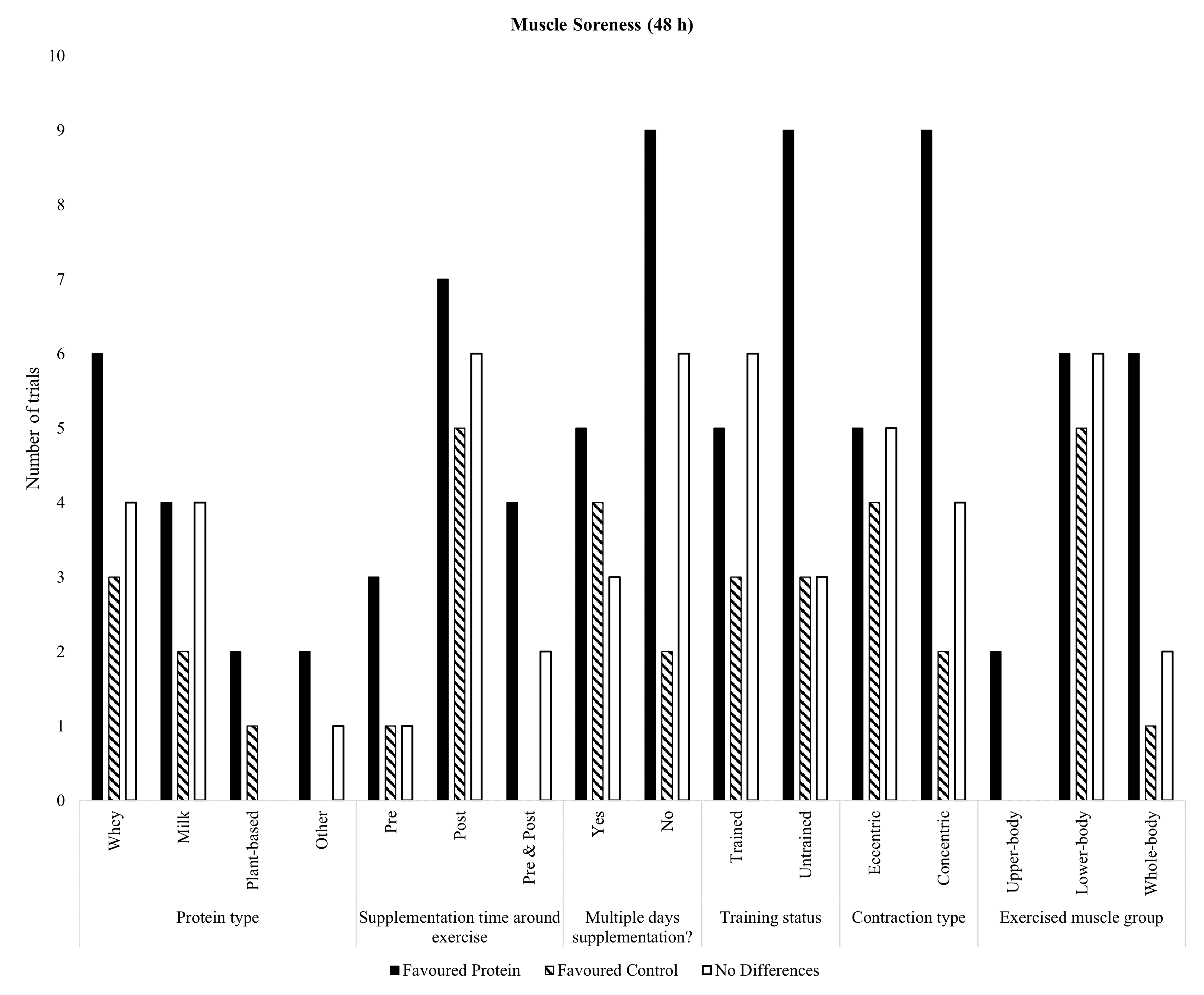


***b***

***Figure S.1*** *Frequency of trials reporting a favourable effect of protein or control supplementation on* ***a.*** *isometric and isokinetic maximal voluntary contraction at 24 h post-exercise categorised by study variables and* ***b.*** *muscle soreness at 48 h post-exercise categorised by study variables*
